# Therapeutical Notes

**Published:** 1899-03-18

**Authors:** 


### THERAPEUTICAL NOTES.

### Acute Dysentery.

Foxwell (*Birmingham Med. Review*, February) quotes Leahy's saline treatment. He saturates seven ounces of water with sulphate of magnesium, and adds one ounce of dilute sulphuric acid. Of this he gives a table-spoonful every one or two hours, and thus treated 45 cases with only three deaths. The de-emetinised ipecacuanha may be given instead of the common drug. In other cases Foxwell gives by the mouth a powder containing morphia grain $\frac{1}{12}$ and calomel grain $\frac{1}{6}$, and he administers every hour an enema of two pints of water with two ounces of boracic acid. After this has been returned a starch enema with a few drops of laudanum is thrown up. Occasional injections of nitrate of silver, 60 grains to the pint, are also very helpful in addition to the antiseptic ones. The bowels should be kept open daily in chronic cases.

### Unna's Ichthyol Ointment for Profuse Sweating of the Feet.

This formula is given as follows in the *International Med. Mag.*, September, from the *Gaz. Hebd. de Med. et de Chirurgie*:—

℞ Ichthyol, 25 parts.
Water, 15.
Lanolin, 25.
M. To be used as an ointment.

